# Telecoaching: a potential new training model for Charcot-Marie-Tooth patients: a systematic review

**DOI:** 10.3389/fneur.2024.1359091

**Published:** 2024-05-09

**Authors:** Ignazio Leale, Vincenzo Di Stefano, Carola Costanza, Filippo Brighina, Michele Roccella, Antonio Palma, Giuseppe Battaglia

**Affiliations:** ^1^Sport and Exercise Research Unit, Department of Psychology, Educational Sciences and Human Movement, University of Palermo, Palermo, Italy; ^2^Ph.D. Program in Health Promotion and Cognitive Sciences, University of Palermo, Palermo, Italy; ^3^Department of Psychology, Educational Science and Human Movement, University of Palermo, Palermo, Italy; ^4^Neurology Unit, Department of Biomedicine, Neuroscience, and Advanced Diagnostics (BiND), University of Palermo, Palermo, Italy; ^5^Department of Sciences for Health Promotion and Mother and Child Care “G. D’Alessandro”, University of Palermo, Palermo, Italy; ^6^Regional Sports School of Italian National Olympic Committee (CONI) Sicilia, Palermo, Italy

**Keywords:** Charcot-Marie-Tooth, adapted physical activity, telecoaching, exercise, neuropathy

## Abstract

**Introduction:**

Charcot–Marie–Tooth disease (CMT) is an inherited neuropathy that affects the sensory and motor nerves. It can be considered the most common neuromuscular disease, with a prevalence of 1/2500.

**Methods:**

Considering the absence of a specific medical treatment and the benefits shown by physical activity in this population, a systematic review was completed using several search engines (Scopus, PubMed, and Web of Science) to analyze the use, effectiveness, and safety of a training program performed in telecoaching (TC). TC is a new training mode that uses mobile devices and digital technology to ensure remote access to training.

**Results:**

Of the 382 studies identified, only 7 met the inclusion criteria. The effects of a TC training program included improvements in strength, cardiovascular ability, and functional abilities, as well as gait and fatigue. However, the quality of the studies was moderate, the size of the participants in each study was small, and the outcome measured was partial.

**Discussion:**

Although many studies have identified statistically significant changes following the administration of the TC training protocol, further studies are needed, with appropriate study power, better quality, and a higher sample size.

## Introduction

CMT also named hereditary motor and sensory neuropathy (HMSN) (1), encompasses a heterogeneous set of disorders affecting peripheral nerves. CMT is the most common inherited neuromuscular disorder with an estimated prevalence of 1 in 2500 (2, 3), caused by mutations in more than 80 genes (4, 5). In this disease, the most common subtypes are CMT1A, CMT1X, hereditary neuropathy with a predisposition to pressure paralysis (HNPP), CMT1B and CMT2A. The other identified subtypes represent less than 1% (6). CMT1 is characterized by a slow conduction speed in the motor nerves caused by a high demyelination process. According to Latour, the CMT1A is the most common form (76%), followed by CMT1X (11%), CMT1B (6%), and CMT1C (<6%) (7). CMT2 is characterized by a mutation in the MFN2 gene, which causes axonal neuropathy with onset in childhood. In general, patients with CMT show a high genetic heterogeneity but with a relatively homogeneous clinical component. Clinically the main symptoms include distal weakness, muscle atrophy, loss of proprioception, sensory loss, reduced reflexes, and skeletal deformities such as hollow foot (8, 9). The combination of these symptoms causes in patients an altered gait associated with difficulty in balance (10–13). It has also been shown that patients with CMT have anxiety and depression problems (14) with alteration of body composition (15) and low levels of physical activity (PA) (14, 16–18). A sedentary lifestyle increases body weight, muscle loss, and musculoskeletal pain, worsening the medical case of patients with CMT (19); this condition increases the risk of comorbidities such as metabolic syndrome or cardiovascular disease (20). The optimization of body composition profiles should be one of the main objectives to be achieved in patients with CMT. In fact, in these patients, a strong relationship between lean mass and physical functionality has been demonstrated (15). In addition, reduced fat mass is related to improved quality of life and reduced symptoms (15).

The current absence of specific medical treatment for several neurological diseases (21), and the need for new evidence for the use of drugs (22) emphasizes the importance of developing sustainable PA programs (8). Indeed, physical exercise might be an effective tool to improve quality of life and improve lifestyle in these patients, as well as slowing the progression of the disease. In particular, aerobic exercises, resistance training, and stretching appear to be the main training methods for CMT patients (23). However, it could be challenging to investigate new types of exercise in these patients, considering the potential benefits of PA but the difficulties in its practice in this population (24). Therefore, this systematic review aims to analyze the effectiveness and safety of TC training programs for CMT patients. Physical exercise in TC involves the use of technological and digital tools, such as computers and mobile devices, to remotely access training content (25).

Physical exercise in TC may be useful to reverse the sedentary lifestyle of CMT patients, allowing them to gain greater autonomy and independence, and improving their quality of life.

## Methods

### Search strategy

This systematic review was developed based on the favored reporting items for systematic reviews and meta-analyses (PRISMA) guidelines (26). The studies were selected through a database screening, such as PubMed (NLM) Web of Science (TS), and Scopus. The following keywords have been included to identify the articles: exercise, physical activity, telecoaching, tele-coaching, Charcot–Marie-Tooth, aerobic training, resistance training, and training. Terms were included in three different groups. In Group A the terms “telecoaching” and “tele-coaching” were inserted, in Group B the terms “exercise,” “physical activity,” “aerobic training,” “resistance training” and “training” were inserted. Finally, in Group C has been inserted the “Charcot–Marie-Tooth” term. To analyze the groups, Boolean operators “AND” and “OR” were used. Examples of matching included: Charcot–Marie-Tooth AND aerobic training OR telecoaching; resistance training AND Charcot–Marie-Tooth. All the founded articles were transferred into the Endnote software (Vers. X20 for Windows 11, © Thomson Reuters).

### Eligibility criteria

All studies that have the following criteria were included in this systematic review: (1) original research with full text written in the English language; (2) all study designs different than reviews, meta-analyses, letters to editors, and theses; (3) studies with training protocol in TC for patients with CMT. No gender differences between males and females were used for exclusion criteria. Articles from gray literature have not been investigated. The PICOS approach (Population, Intervention, Comparator, Outcomes, Study design) is explained in Table 1.

**Table 1:** Inclusion criteria according to the PICOS approach.

Category	Details
Population	Males and female subjects with Charcot–Marie–Tooth disease
Intervention	Physical endurance, physical fitness, aerobic training, resistance training, and physical therapy performed in telecoaching.
	Training protocols not performed in TC have been excluded.
Comparator	No restriction
Outcome	Muscle strength, aerobic capacity, balance, psychological status, quality of life, physiological adaptations, or functional ability.
Study design	Randomized clinical trials, randomized controlled trials, or clinical trials. Articles that were not published in the last 10 years, systematic reviews, scoping reviews, meta-analyses, letters to editors, and grey literature have been not investigated.

### Study selection

The identification and elimination of duplicate studies were carried out by a single researcher. Subsequently, two authors analyzed independently the articles. Initially, the authors analyzed the title and abstracts of each study. Secondly the authors analyzed the full text to assess whether the selected studies agreed with the criteria of inclusion and exclusion.

The two authors discussed any dispute regarding their decisions; if the two authors did not agree on the inclusion or exclusion of a study, a third auditor was consulted. A Microsoft Excel spreadsheet (Microsoft Corp, Redmond, Washington) was used to report the following information related to the included studies: year of publication, sample age, gender, the aim of the study, and the TC protocol.

### Quality assessment

Two authors evaluated the quality and bias of all the studies included in this review, using a modified version of the checklist to measure the quality of RCTs and not RCTs written by Downs and Black (27). In the modified version of the checklist, item 27 (power) was changed from 0 to 5 to a score of 0–1, so that a study would receive a score of 0 in case the statistical power was below 80%, a score of 1 if the statistical power was above 80% (28). As a result, the final checklist score changed from 32 to 28. The quality of the studies was divided into four levels: excellent (26–28), good (20–25), fair (15–19), and poor (<14) (29, 30). The authors’ results were compared by an external researcher and discrepancies were resolved in a consensus meeting.

## Results

### Study identification

**Figure 1:**
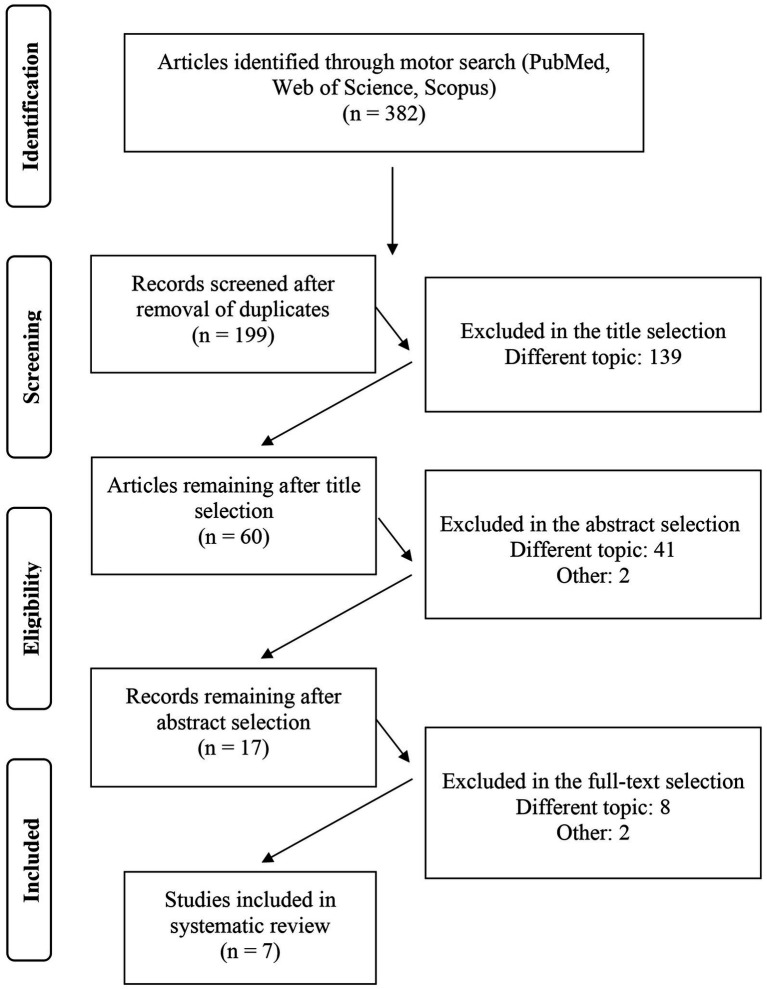
Flow diagram representing the selection process of manuscripts.

A total of 382 studies were identified through electronic databases. The titles and abstracts of 199 studies were analyzed after removing 183 duplicates. The full text of 17 studies has been examined of which only 7 articles agree with the inclusion and exclusion criteria. The PRISMA flowchart shows the entire selection process (Figure 1).

In detail, of the 7 included studies, 5 analyzed the effectiveness of a resistance training protocol in patients with CMT (31–35), while 2 studies analyzed the effectiveness of an interval training protocol on this target population (36, 37).

A total of 170 patients with CMT were included in this review with an age between 11 and 45 years. Of the 7 studies, it is possible to identify two studies of El Mhandi et al. (36, 37) that included only male participants. The other five studies included both male and female participants. More detailed information on each study can be found in Table 2.

**Table 2:** The aim of the manuscripts and relative characteristics of the sample.

1st author, year	Participants [f] [m], Age ± SD	Aim	Telecoaching
Burns, 2017	60 [30] [30], 11, 5 ± 3, 3	Evaluate the safety and efficacy of progressive resistance exercise for foot dorsiflexion weakness in children with CMT disease.	12/72 supervised training sessions. A demonstration video for each participant. An exercise diary. Parents as a guide.
Chetlin, 2004	20 [11] [9], 45, 2 ± 8, 9	Evaluate the effects of a home-based resistance exercise program on strength, body composition, and activities of daily living in patients with CMT disease.	Weekly telephone calls. A demonstration video for each participant. An exercise diary.
Chetlin, 2004	20 [11] [9], 45, 2 ± 8, 9	Evaluate the effects of resistance training with and without creatine supplementation on muscle, strength, and function in patients with CMT disease.	Weekly telephone calls. A demonstration video for each participant. An exercise diary.
El Mhandi, 2008	8 [0] [8], 34 ± 11	Evaluate if 24 weeks of interval-training exercise cycling can significantly improve physiological, neuromuscular, and functional capacities and alleviate fatigue in CMT patients.	Weekly telephone calls. A heart rate monitor for each participant. An exercise diary.
El Mhandi, 2008	16 [0] [16], 33 ± 8	Evaluate the effects of an interval exercise training program on heart rate variability in CMT patients.	Weekly telephone calls. A heart rate monitor for each participant. An exercise diary.
Ramdharry, 2014	32 [17] [15], 44 ± 15	Evaluate the effects of a 16-week home-based program of resistance training on hip flexor muscle strength in CMT patients.	Weekly telephone calls. Monthly visits to progress resistance. An exercise diary.
Dudziec, 2023	14 [11] [3], 44 ± Na	Evaluate the effects of a resistance training program and multi-modal program of proprioceptive rehabilitation performed at home on the ability to balance.	3 supervised training sessions. Weekly telephone calls.

F, female; M, male; SD, standard deviation; CMT, Charcot–Marie–Tooth disease; Na, not available.

### Methodological quality

The methodological quality of the included studies was analyzed using the modified version of the Downs and Black checklist. Out of 7 studies, 1 study was evaluated as “good quality” (34), 3 studies as “fair quality” (31, 33, 35), and 3 studies were evaluated as “poor quality” studies (32, 36, 37). Some items of Downs and Black are difficult to use in studies with physical activity protocol, such as the use of a study design in double-blind.

### Resistance training

Of the extrapolated studies, five used a TC resistance training protocol in patients with CMT (31–34). Among these, there is the study of Burns and colleagues (31); a randomized, double-blind, sham-controlled trial, in which the experimental group performed a TC training resistance exercise, while the control group performed a fictitious workout. The treatment team was the only one who knew about group assignment; participants, parents, caregivers, clinical evaluators, and other investigators were not informed of the group allocation. The experimental group, consisting of children, had performed a TC training program that aimed to improve the weakness in the dorsiflexion of the foot. Participants were trained using an adjustable ankle bracelet, designed to prevent slipping, three times a week or non-consecutive days, for 6 months with a protocol that provided an initial intensity of 50% and a progressive increase of up to 70% of one repetition maximum (1RM). The control group trained with the same procedure, including the same equipment, the same duration, the same frequency, the same series, and repetitions. However, the participants trained with a very low intensity, never exceeding 10% 1RM. Each parent was asked to help the child with the application of the bracelet and to supervise the training performed in TC. TC included the compilation of an exercise diary, the delivery of a video to be used as a familiarization, and the active participation of parents as a guide, as well as the execution of some supervised training sessions. The results indicated that after a 24-week intervention period, the experimental group had reduced the long-term progression of dorsiflexion weakness without side effects on muscle morphology; there were no signs of overwork fatigue. It has been shown that a TC training program is safe, valid, and effective in a pediatric population affected by CMT disease. A drop-out of 5 patients was detected, demonstrating high adherence to the training mode. Also Chetlin’s study (34) used a home-based resistance training program, for 12 weeks. This study aimed to evaluate the efficacy of resistance training with and without creatine supplementation on muscle strength and activities of daily living (ADL) in patients with CMT disease. ADL included getting up from the chair without using your arms, getting up from a supine position, and going up and down the stairs. The subjects were also timed while performing a lifting and moving test. Each test was performed from 3 to 6 times. In detail, 20 patients with CMT consumed 5 g/day creatine or placebo, while participating in a resistance training program. Resistance training consisted of 12 weeks of exercise performed in TC, divided into three phases (each phase of 4 weeks) with the amount of work increased systematically by adjusting the resistance or repetitions for each of the phases. For example, hand-grip exercises consisted of 3 sets of 4 repetitions, three times per week, with a 1-min rest period between each set. If the subjects failed to perform the number of repetitions, they used a lower resistance to complete the required program. More details can be found in Table 3. TC included the delivery of videos describing in detail the training techniques as well as supervised training sessions in which subjects knew the exercises to be performed.

**Table 3:** Type of treatment and protocol in telecoaching.

Author, Years	Exercise	Intervention (*n*)	Treatment (time)	Training protocol
Burns, 2017	RT	TCG (30) vs. CG (30)	24 weeks of training, 3 times/week	TCG: exercises with an intensity that is initially at 50% and progressed to 70% of the 1RM. CG: participants trained at very low intensity (<10% of one repetition maximum).
Chetlin, 2004	RT	Male TCG (11) vs. Female TCG (9)	2 weeks of training, 3 times/week	TCGs: Resistance training was increased as follows: knee flexors and extensors, 40% (phase I), 45% (phase II), 50% (phase III); elbow flexors and extensors, 20% (phase I), 25% (phase II), 30% (phase III). Repetitions: week 1, 4RP; week 2, 6 RP; week 3, 8 RP; and week 4, 10 RP.
Chetlin, 2004	RT	TCG (10) vs. TCG+ integration (10)	12 weeks of training, 3 times/week	TCGs: Resistance training was increased as follows: knee flexors and extensors, 40% (phase I), 45% (phase II), 50% (phase III); elbow flexors and extensors, 20% (phase I), 25% (phase II), 30% (phase III). Repetitions: week 1, 4RP; week 2, 6 RP; week 3, 8 RP; and week 4, 10 RP. Integration: 5 g creatine monohydrate fortified with 2 g dextrose (Neotine; Avicena, Cambridge, MA) daily.
El Mhandi, 2008	ITE	TCG group (8)	24 weeks of training, 3 times/week	TCG: cycling program adapted; 5-min warm-up followed by six 5-min exercise bouts: 4 min at 40% of Pmax alternated with 1 min at 80% of Pmax.
El Mhandi, 2008	ITE	TCG (8) vs. CG (8)	24 weeks of training, 3 times/week	TCG: 5-min warm-up followed by six 5-min exercise bouts: 4 min at 40% of Pmax alternated with 1 min at 80% of Pmax. CG: No intervention.
Ramdharry, 2014	RT	TCG (18) vs. CG (14)	16 weeks of training, 4 times/week	TCG: Ankle weights with 0.5 kg increments and progressed from loads at 40% of the maximal voluntary isometric contraction to 60%. Two consecutive sets of 8–12 repetitions. CG: No intervention.
Dudziec, 2023	RT	TCG (7) vs. CG (7)	12 weeks of training, 4 times/week	TCG: Individualized, proximal lower limb and trunk, body weight-based, resistance exercises were performed on alternate days. Postural exercises included mechanical and sensory stimuli. CG: No intervention

CG, control group; TCG, telecoaching group; CMT, Charcot–Marie–Tooth disease; RT, resistance training; ITE, interval-training exercise; RP, repetitions; ADLs, activities of daily living; HRV, Heart Rate Variability; Pmax, maximal aerobic power.

The results indicated that for both groups, the type I muscle fiber diameter, and strength, were improved as well as the ADL. Overall, patients with CMT respond to resistance training performed in TC with muscle fiber adaptations, and with improvements in strength, and in the ADL. However, the use of Creatine was not beneficial. No drop-out has been detected.

The same research design has been applied by Chetlin and colleagues (32) to determine the effectiveness of a TC resistance training program on body composition, strength, and function of daily activities in men and women with CMT. The women’s group consisted of 11 participants while the men’s group of 9. At baseline, women had a normal percentage of strength (80%) in 4 out of 10 measurements, while men did not reach 80% of normal force in any measure. After resistance training, women reached 80% of normal strength in 8 out of 10 measurements, while men in 1 measurement. The functions of daily activities were improved after training without gender differences; moreover, men had significantly more fat-free mass (*p* < 0.05) before and after training compared to women.

Ramdharry and colleagues (33) used a randomized single-blind crossover design to assess the effectiveness of a physical exercise in TC on hip flexor muscle strength. Randomization was used to assign the subjects to one of two groups: group A with initial training, and group B with initial control period. The training protocol conformed with the guidelines of the American College of Sports Medicine (ACSM) and provided 16 weeks of training with 4 weekly training (38). Weights were used, positioned in the ankle, with periodic increments of 0.5 kg and oscillation from 40 to 60% of the maximum voluntary isometric contraction (MVC). Participants performed 2 sets of 8–12 repetitions, 4 days a week for 16 weeks. The exercise was performed in the supine position with load displacement from 10° in hip extension to 45° in hip flexion. After a period of familiarization, patients were monitored through an exercise diary, weekly phone calls, and monthly visits to improve protocol adherence. The results showed a significant increase in the strength of the hip flexor muscles to the left but not to the right, demonstrating that the resistance training was well-tolerated without evidence of weakness from overload. In addition, no drop-out was recorded, indicating high compliance with this training mode.

Finally, Dudziec and colleagues (35) used TC training protocol to assess the effectiveness of resistance training and multisensory balance training, delivered at home, on the ability to balance in patients with CMT. 14 patients were randomized into two groups: the TC group performed 12 weeks of strength training and multisensory balance; the control group performed the daily activities. The results showed high adherence to this training modality with 93% of patients completing the study. Posturography tests showed improvement in postural stability in favor of the intervention group, and the ability to balance and gait showed an improved elevated in the TC group. In the same group, partial improvements have been shown in the strength of the lower limbs.

### Interval-training exercise

Only two of the studies analyzed have used a TC protocol of interval training. El Mhandi and colleagues (37) have used this training program to assess if 24 weeks of interval training cycles could significantly improve physiological, neuromuscular, and functional abilities, as well as alleviate the perception of fatigue in patients with CMT. The training program included 24 weeks of training of which 12 were supervised and 12 telecoaching-independently at home. The single training session was 45 min and included 5 min of warm-up, 30 min of the middle phase, and 10 min of cool-down. No stretching activity was foreseen. The central phase consisted of 6 exercises of 5 min, of which 4 min at 40% of the maximum aerobic power (Pmax) and 1 min at 80% of Pmax. The exercise was made on stationary cycloergometers. TC period was performed with the same protocol, although several personal cycloergometers were used by subjects in their homes. Subjects were motivated and checked by weekly phone calls. The training period was well tolerated; no subjects reported adverse events or complications during the protocol. The results showed significant improvements in cardiorespiratory abilities, functional ability, and isokinetic strength, as well as in the scale of pain and fatigue during training. Also, benefits obtained at the end of the supervised period were maintained after the home period. However, the results also revealed that there was no effect on the production of isometric strength and indices of fatigue resistance after training.

The same training protocol was applied by El Mhandi and colleagues (36) to assess the effectiveness of interval training on heart rate variability (HRV) in patients with CMT. An experimental group and a healthy control group were included in this study. The HRV assessment was conducted at the baseline (T0) and the end of the 24-week study period (T1); in both evaluations, subjects were monitored through 24-h Holter electrocardiographic (ECG). The results showed that at the baseline all HRV indices of the experimental group were lower than the control group with a statistically significant difference for the pNN50 parameter. After the training program, the researchers found improvement in some time and frequency parameters in the experimental group; in some cases, the values of the experimental group at T1 exceeded the values of the control group at baseline (+8% mean R–R interval, +95% pNN50, 52% reduction in low/high-frequency ratio). These results suggest that interval training might improve dysautonomia in CMT, improving the activity of the parasympathetic nervous system.

## Discussion

The present review aimed to analyze the effectiveness of TC programs in patients with CMT disease. The topic is innovative and punctual, but there are still few studies in the literature. It has been found that this modality of exercise training can determine significant improvements in motor strength, functional abilities, heart rate variability, activities of daily life, as well as cardiorespiratory ability. However, due to the low number of randomized controlled trials, the low quality of the studies, the reduced number of participants within each training protocol, and the limited evidence of pre-post differences, the true effectiveness of TC in CMT disease remains unclear. Most of the studies analyzed examined the effectiveness of a TC resistance training program, showing positive changes in the indices of strength and muscle weakness, associating them with an increase in physiological abilities, neuromuscular and functional. Similarly, interval training showed significant improvements in HRV and the perception of fatigue, as well as an increase in cardiovascular ability.

In detail, Burns and colleagues (31) showed that 6 months of resistance training in TC preserved the strength of the dorsiflexion in the children; while over the weeks, likewise of an adult with CMT (39, 40), children have produced through resistance training, an overall gain of strength of 5% compared to a deterioration of 23% for the sedentary children, reflecting the normal course of the disease (41). Although creatine has not involved any more significant changes, the improvements induced by the training protocol of Chetlin and colleagues (32, 34), show the effectiveness of the TC training mode. The results obtained could be the result of muscle adaptation induced by resistance training. Indeed, post-workout, a hypertrophy of type 1 fibers was found, a condition consistent with what happens in healthy people after high-volume training (42). However, subjects with CMT are not able to train at the same intensities recommended for healthy sedentary subjects (8–12 repetitions to exhaustion or 67–80% of maximum strength) (43). Considering that the high intensity of training is associated with a decrease in performance and an increased risk of injury in patients with neuromuscular diseases (44), CMT patients needed to perform more repetitions, with less intensity, to equalize the recommended training volume. About creatine, it has been shown that oral intake increases muscle levels of creatine and phosphocreatine in healthy subjects, improving performance and post-exercise recovery (45–47). In detail, subjects who increase creatine levels by 17–30% get the biggest increases (48), while those who increase by 5% do not get any performance improvement (49). Conversely, although the initial levels of creatine are within the reference values for subjects with neuropathy (50), the lack of benefits induced by taking it might be caused by a low percentage of intake (5%).

Not increasing muscle creatine concentration in patients with CMT may be caused by the non-compliant use of the supplement or an alteration of the creatine transporter. Future studies could analyze the correct functioning of the creatine transporter in patients with CMT.

Moreover, the study by Ramdharry and colleagues (33), showed contrasting results, demonstrating a modest improvement in hip strength, but only to the left side. Overall, resistance training performed in TC would seem beneficial for this population, even if in the programming phase it should be considered that the muscles of CMT subjects do not physiologically respond to the training session due to chronic denervation (51). In addition, the sedentary lifestyle of CMT patients may be the cause of the improvements achieved by resistance training evidenced in other studies, as seen in healthy subjects (52); hence, it could be useful to extend the protocol administration period. Also, the protocol of El Mhandi and colleagues (36, 37) of interval training performed in TC was an effective strategy, aligning with other studies that demonstrate the effectiveness of regular and progressive dynamic activity in neuromuscular diseases (53–56). The numerous positive results obtained show that the program developed by researchers is perfectly in line with the needs and abilities of patients with CMT. A program that requires high functional abilities may be difficult to integrate into the daily routine, demotivating patients and causing a high risk of abandonment (57). A physical activity program should consider this aspect, especially if executed in TC; indeed, it has been shown that poor motivation becomes an obstacle to exercise for both the patient and the healthcare professional (58, 59). Moreover, recruitment is one of the main problems of studies conducted on CMT (60), because many patients with neuromuscular diseases do not undertake a physical activity program for lack of motivation and difficulties in shifts and logistics. Indeed, only one of the studies in this review has analyzed the sample size to ensure sufficient power in its study, to detect a statistically significant effect (33); the remaining studies did not use any reference to sample power. From this perspective, TC could be a training mode that helps this population by offering a practical and attainable approach. In addition, physical exercise in TC for patients with CMT should consider the age of participants. All studies included recruited adult participants with an average age of 40 years, except for the study conducted by Burnsand colleagues (31), in which participants were children between the ages of 6 and 17 years and an average age of 11.5 years. The age of the participants could influence both recruitment and results obtained; it is more likely that the main benefits induced by physical exercise in TC, are obtained at the disease onset, therefore in the early stages of the disease when the clinical burden is limited, and chronic complications have still not overcome.

### Future directions

A training program performed in TC has already been used in many other target populations, resulting in effective and risk-free (61, 62). From the analysis of these studies, it is possible to observe the main strengths and limitations of this new training approach. Indeed, the execution of physical exercise in TC has several advantages, among these we can identify the reduction of economic resources, the reduction of geographical limits, the freedom to train when you want as well as the possibility of training in a family environment and safe. In addition, this mode increases the autonomy and independence of the patient. However, TC has also negative factors, including social isolation, loss of contact between coach and patient, the high risk of abandonment, and the difficulty for the patient to understand the right execution technique of the exercises.

For these aspects, physical exercise in TC requires telecoaches flexible and available to every need of the patient, constant weekly calls in order to supervise and control the progress of the exercise as well as an initial familiarization in order to learn and improve the execution of the exercises. Moreover, to optimize the effectiveness of this training modality, could be useful to provide each patient a clear and concise digital material that explains in detail the exercises to be performed, such as demo videos. Another important tool could be an exercise diary, in which to record when the training is carried out and any personal considerations in order to optimize PA.

## Conclusion

Overall, this systematic review has found consistent results regarding the effectiveness and safety of a training protocol performed in TC, resulting in free of adverse events, with high adherence to the training mode and effective on all the variables analyzed. However, due to the low number of randomized controlled trials, the low quality of the studies, the reduced number of participants within each training protocol, and the limited evidence of pre-post differences, the true effectiveness of TC in CMT disease is still unclear. The TC training intervention allows researchers to develop new research strategies. Considering this, a multicenter study project involving a larger number of subjects could be the main intervention strategy to reduce these limits.

Further research is needed to verify the real benefits of a training protocol in TC and to organize and detail the training parameters such as volume, intensity, and density.

## Data availability statement

The original contributions presented in the study are included in the article/supplementary material, further inquiries can be directed to the corresponding author.

## Author contributions

IL: Conceptualization, Formal analysis, Methodology, Writing – original draft. VS: Conceptualization, Formal analysis, Methodology, Writing – original draft. CC: Formal analysis, Writing – original draft. FB: Supervision, Writing – review & editing. MR: Writing – review & editing. AP: Supervision, Writing – review & editing. GB: Conceptualization, Methodology, Supervision, Writing – review & editing.
